# Year-Long Stability of Nucleic Acid Bases in Concentrated Sulfuric Acid: Implications for the Persistence of Organic Chemistry in Venus’ Clouds

**DOI:** 10.3390/life14050538

**Published:** 2024-04-23

**Authors:** Sara Seager, Janusz J. Petkowski, Maxwell D. Seager, John H. Grimes, Zachary Zinsli, Heidi R. Vollmer-Snarr, Mohamed K. Abd El-Rahman, David S. Wishart, Brian L. Lee, Vasuk Gautam, Lauren Herrington, William Bains, Charles Darrow

**Affiliations:** 1 Department of Earth, Atmospheric and Planetary Sciences, Massachusetts Institute of Technology, Cambridge, MA 02139, USA; jjpetkow@mit.edu (J.J.P.); laurenis@mit.edu (L.H.);; 2 Department of Physics, Massachusetts Institute of Technology, Cambridge, MA 02139, USA; 3 Department of Aeronautical and Astronautical Engineering, Massachusetts Institute of Technology, Cambridge, MA 02139, USA; 4 Nanoplanet Consulting, Concord, MA 01742, USA; 5 JJ Scientific, 02-792 Warsaw, Poland; 6 Faculty of Environmental Engineering, Wroclaw University of Science and Technology, 50-370 Wroclaw, Poland; 7 Department of Chemistry and Biochemistry, Worcester Polytechnic Institute, Worcester, MA 01609, USA; 8 Complex Carbohydrate Research Center, University of Georgia, 315 Riverbend Road, Athens, GA 30602, USA; jgrimesjr@uga.edu; 9 Department of Chemistry and Chemical Biology, Harvard University, Cambridge, MA 02138, USAhrvsnarr@fas.harvard.edu (H.R.V.-S.);; 10 Department of Biological Sciences, University of Alberta, Edmonton, AB T6G 2E9, Canada; 11 Department of Computing Science, Faculty of Pharmacy and Pharmaceutical Studies, University of Alberta, Edmonton, AB T6G 2H1, Canada; 12 Department of Laboratory Medicine and Pathology, Faculty of Pharmacy and Pharmaceutical Studies, University of Alberta, Edmonton, AB T6G 2H1, Canada; 13 School of Physics and Astronomy, Cardiff University, 4 The Parade, Cardiff CF24 3AA, UK; 14 Rufus Scientific, Melbourn, Herts SG8 6ED, UK

**Keywords:** Venus, NMR, nucleic acid bases, sulfuric acid

## Abstract

We show that the nucleic acid bases adenine, cytosine, guanine, thymine, and uracil, as well as 2,6-diaminopurine, and the “core” nucleic acid bases purine and pyrimidine, are stable for more than one year in concentrated sulfuric acid at room temperature and at acid concentrations relevant for Venus clouds (81% *w*/*w* to 98% *w*/*w* acid, the rest water). This work builds on our initial stability studies and is the first ever to test the reactivity and structural integrity of organic molecules subjected to extended incubation in concentrated sulfuric acid. The one-year-long stability of nucleic acid bases supports the notion that the Venus cloud environment—composed of concentrated sulfuric acid—may be able to support complex organic chemicals for extended periods of time.

## 1. Introduction

Venus has a surface that is too hot for any plausible solvent and the most complex organic chemistry; hence, it is unsuitable for supporting life. Nevertheless, scientists have speculated that the perpetual cloud cover at 48 to 60 km above Venus’ surface, and with temperatures matching those found at Earth’s surface, might host life (see, e.g., [1,2,3,4,5,6,7,8,9]). Venus clouds, however, are composed of concentrated sulfuric acid—an aggressive solvent that destroys most of Earth life’s biochemicals and is thought to be sterile to complex organic chemistry or life of any kind. Here, we build on early, decades-old reports on the reactivity of a few purines and pyrimidines in concentrated sulfuric acid (e.g., [10,11,12,13]), as well as on our previously published work [14], to explore the long-term stability of the key molecules needed for life. We study the year-long stability of nucleic acid bases at room temperature at acid concentrations relevant for Venus clouds (81% *w*/*w* to 98% *w*/*w* sulfuric acid, the rest water).

## 2. Materials and Methods

The materials, methods, and procedures are the same as those in our initial study [14], so we provide only a brief summary here. To acquire NMR data, we used a Bruker Avance III-HD 400 MHz spectrometer equipped with a Prodigy liquid nitrogen cryoprobe (BBO) at 25 °C. We incubated 30–40 mg of each nucleic acid base in 81–98% *w*/*w* D_2_SO_4_ with the rest D_2_O for one year. After one year of incubation (Table 1, Table 2, Table 3, Table 4, Table 5, Table 6, Table 7 and Table 8; measurements done in November 2023), we measured 1D ^13^C and 1D ^1^H NMR spectra at each of the tested acid concentrations and compared them to the original NMR spectra collected after ~30–48 h (Table 1, Table 2, Table 3, Table 4, Table 5, Table 6, Table 7 and Table 8; measurements done in October 2022). The NMR tubes with solutions of nucleic acid bases dissolved in different concentrations (by weight) of sulfuric acid in water (98% D_2_SO_4_/2% D_2_O; 94% D_2_SO_4_/6% D_2_O; 88% D_2_SO_4_/12% D_2_O; 81% D_2_SO_4_/19% D_2_O) were stored at room temperature without any protection from light for over a year before the ^1^H and ^13^C NMR measurements were taken.

We used MNova software (Mestrelab Research) to process and analyze the NMR data [15]. The original data for all NMR experiments are available for download from Zenodo at https://zenodo.org/records/10793625 (accessed on 7 March 2024).

## 3. Results: Nucleic Acid Bases Are Stable in Concentrated Sulfuric Acid for at Least a Year

**Figure 1:**
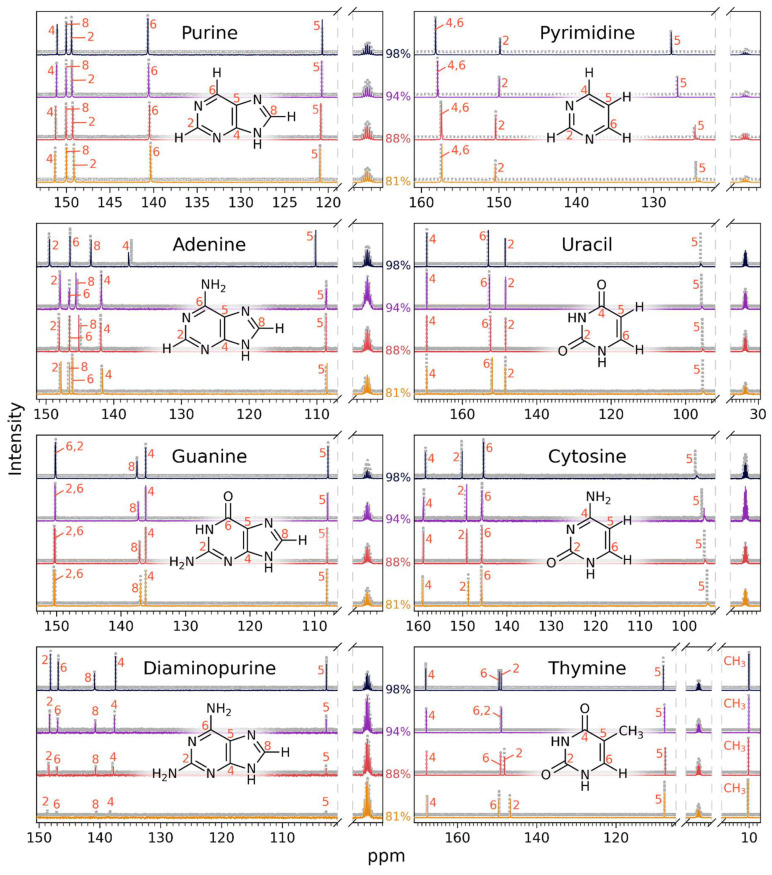
Comparison of 1D ^13^C NMR spectra of nucleic acid bases and related molecules incubated in concentrated sulfuric acid for two different time periods. The intensity (*y*-axis) is shown as a function of spectral shift in parts per million (ppm) (*x*-axis). Each compound’s NMR spectrum is shown in an individual subfigure. Within each subfigure, we compare the NMR spectrum of the year-long incubation (colored lines) to the NMR spectrum collected after ~30–48 h (dashed grey line spectra from [14]). For clarity, the ~30–48 h spectra are displayed with a slight vertical offset. From top to bottom within each subpanel, we show the NMR spectra of compounds dissolved in different concentrations (by weight) of sulfuric acid in water: 98% D_2_SO_4_/2% D_2_O; 94% D_2_SO_4_/6% D_2_O; 88% D_2_SO_4_/12% D_2_O; 81% D_2_SO_4_/19% D_2_O with DMSO-d_6_ as a reference and at room temperature. All peaks are consistent, with the molecules being stable and the structure not being affected by the concentrated sulfuric acid solvent. The one-year spectra and the ~30–48 h spectra look virtually identical for all tested concentrations, demonstrating the year-long stability of the compounds in the concentrated sulfuric acid solvent.

**Figure 2:**
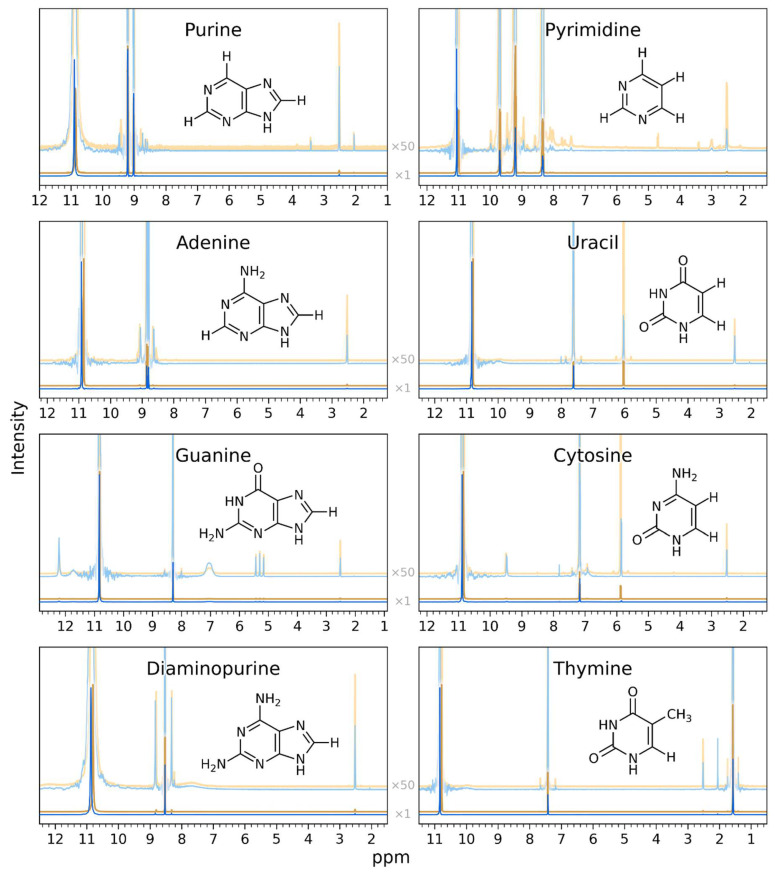
Comparison of 1D ^1^H NMR spectra of nucleic acid bases and related molecules in concentrated sulfuric acid for two different time periods. The intensity (*y*-axis) is shown as a function of the spectral shift in parts per million (ppm) (*x*-axis). Each compound’s NMR spectrum is shown in an individual subfigure. Within each subfigure, we compare the NMR spectrum of the year-long-incubation (blue foreground spectra) to the NMR spectrum collected after ~30–48 h (orange background spectra from [14]). For clarity, the ~30–48 h spectra are displayed with a slight vertical offset. From top to bottom within each subpanel, we show the NMR spectra of compounds dissolved in 98% D_2_SO_4_/2% D_2_O (by weight) with DMSO-d_6_ as a reference and at room temperature. Within each subfigure, each spectrum is plotted twice: once using “normal” scaling and once multiplied vertically by a factor of 50 to reveal low-intensity features (light-colored zoomed-in spectra). The large peak around 11 ppm corresponds to the D_2_SO_4_ solvent. All peaks are consistent, with the molecules being stable and the structure not being affected by the concentrated sulfuric acid solvent. The one-year spectra and the ~30–48 h spectra look virtually identical, demonstrating the year-long stability of the compounds in a concentrated sulfuric acid solvent.

We demonstrate the one-year-long stability of nucleic acid bases in concentrated sulfuric acid by comparing the ^13^C NMR and ^1^H NMR spectra collected after ~30–48 h of incubation from our previous study [14] to the ^13^C NMR and ^1^H NMR spectra collected for the same samples after a one-year long incubation in concentrated sulfuric acid (Figure 1 and Figure 2). The spectra of the one-year-old sample and the ~30–48 h old sample overlap perfectly and look virtually identical for all tested nucleic acid bases in all tested sulfuric acid concentrations, with no signs of reactivity. The compounds in our study are the nucleic acid bases adenine, cytosine, guanine, thymine, and uracil, as well as 2,6-diaminopurine, and the “core” nucleic acid bases purine and pyrimidine under conditions of concentrated sulfuric acid at room temperature and at acid concentrations relevant for Venus clouds (81% *w*/*w* to 98% *w*/*w* acid, the rest water).

For each compound, the number of carbon peaks and their chemical shift position in the ^13^C NMR spectrum and the number of carbon atoms of the original compound are preserved over the span of a one-year-long incubation in 81% *w*/*w* to 98% *w*/*w* sulfuric acid. This result confirms that the aromatic ring structure of the nucleic acid bases remains intact after one year (Figure 1; Table 1, Table 2, Table 3, Table 4, Table 5, Table 6, Table 7 and Table 8).

We note that, over time, the ^13^C NMR signal corresponding to the C5 carbon in pyrimidine, cytosine, and uracil splits and broadens (Figure 1). The splitting and broadening of the C5 peak indicates an efficient exchange of the C5 proton of the pyrimidine ring with the solvent’s deuterium (i.e., H/D exchange) and is not a sign of an instability of the pyrimidine ring. Such an H/D exchange is known to happen readily in acidic solutions [16].

## 4. Discussion

From a chemical point of view, the stability of nucleic acid bases in concentrated sulfuric acid is not surprising. Nucleic acid bases have a basic character and form sulfate salts upon dissolution in sulfuric acid. Indeed, early studies from decades ago on the reactivity of a few purines and pyrimidines in concentrated sulfuric acid had shown that dissolved sulfate salts of nucleic acid bases are very stable to solvolysis in concentrated H_2_SO_4_ (e.g., [10,11,12,13]). Such chemical knowledge, however, rarely crosses disciplines and is not widely recognized in the field of planetary science. Meanwhile, the assessment of the stability and reactivity of life’s chemical building blocks in concentrated sulfuric acid is critical for the true understanding of the habitability of Venus and Venus-like exoplanets.

Our study is the first to test the reactivity and structural integrity of organic molecules subjected to extended incubation in concentrated sulfuric acid. The few past stability studies of organic molecules in concentrated sulfuric acid involved the incubation of the tested substance for hours, days, and weeks, with only a few compounds incubated for a few months (reviewed in [17,18]). We are not aware of any year-long (or other long-term) stability studies of organic molecules in concentrated sulfuric acid. Such long-term stability studies of molecules are valuable not only for organic chemistry but also for the proper assessment of the possibility of the long-term persistence of organics in the clouds of Venus. Several nucleic acid bases (including adenine, guanine, cytosine, thymine, and uracil) have been identified in meteoritic material [19], which suggests a continuous supply of these compounds to Venus’ atmosphere. Therefore, a small but steady supply of nucleic acid bases could be delivered via meteoritic infall to Venus clouds, where they can persist dissolved in cloud particles for many months if not years [8]. Finally, we note that the meteoritic material could also deliver minerals with a catalytic activity that, combined with Venusian cloud chemistry, could promote chemical reactions of organic molecules dissolved in cloud particles (e.g., [20,21]).

## Figures and Tables

**Table 1:** Comparison of ^13^C NMR chemical shifts in guanine incubated in concentrated sulfuric acid for two different time periods. All NMR data were obtained at room temperature.

Guanine										
^13^C										
Solvent	C2 (ppm)		C4 (ppm)		C5 (ppm)		C6 (ppm)		C8 (ppm)	
	*11 October 2022*	*6 November 2023*	*11 October 2022*	*6 November 2023*	*11 October 2022*	*6 November 2023*	*11 October 2022*	*6 November 2023*	*11 October 2022*	*6 November 2023*
81% D_2_SO_4_/19% D_2_O	150.36	150.37	136.33	136.34	108.22	108.22	150.55	150.56	137.11	137.10
88% D_2_SO_4_/12% D_2_O	150.28	150.28	136.26	136.27	108.13	108.14	150.42	150.44	137.22	137.21
94% D_2_SO_4_/6% D_2_O	150.23	150.23	136.22	136.22	108.01	108.01	150.26	150.28	137.38	137.36
98% D_2_SO_4_/2% D_2_O	150.13	150.12	136.17	136.18	107.90	107.90	150.19	150.22	137.53	137.53

**Table 2:** Comparison of ^13^C NMR chemical shifts in cytosine incubated in concentrated sulfuric acid for two different time periods. All NMR data were obtained at room temperature.

Cytosine								
^13^C								
Solvent	C2 (ppm)		C4 (ppm)		C5 (ppm)		C6 (ppm)	
	*14 October 2022*	*6 November 2023*	*14 October 2022*	*6 November 2023*	*14 October 2022*	*6 November 2023*	*14 October 2022*	*6 November 2023*
81% D_2_SO_4_/19% D_2_O	141.98	142.05	152.32	152.38	88.11	88.16	139.06	139.07
88% D_2_SO_4_/12% D_2_O	149.10	149.08	158.95	158.93	95.50	95.45	145.79	145.71
94% D_2_SO_4_/6% D_2_O	149.40	149.17	158.79	158.94	96.06	95.58	145.67	145.70
98% D_2_SO_4_/2% D_2_O	150.23	150.09	158.34	158.37	97.48	97.25	145.28	145.26

**Table 3:** Comparison of ^13^C NMR chemical shifts in diaminopurine incubated in concentrated sulfuric acid for two different time periods. All NMR data were obtained at room temperature.

Diaminopurine										
^13^C										
Solvent	C2 (ppm)		C4 (ppm)		C5 (ppm)		C6 (ppm)		C8 (ppm)	
	*14 October 2022*	*7 November 2023*	*14 October 2022*	*7 November 2023*	*14 October 2022*	*7 November 2023*	*14 October 2022*	*7 November 2023*	*14 October 2022*	*7 November 2023*
81% D_2_SO_4_/19% D_2_O	141.90	— ^1^	131.59	— ^1^	96.10	— ^1^	140.39	— ^1^	133.84	— ^1^
88% D_2_SO_4_/12% D_2_O	148.51	148.43	137.97	137.84	102.93	102.89	147.13	147.07	140.74	140.75
94% D_2_SO_4_/6% D_2_O	148.28	148.30	137.62	137.65	102.83	102.84	146.96	146.98	140.79	140.79
98% D_2_SO_4_/2% D_2_O	148.08	148.11	137.38	137.41	102.74	102.75	146.80	146.82	140.87	140.86

^1^ The spectra could not be collected due to poor solubility of diaminopurine in 81% *w*/*w* sulfuric acid.

**Table 4:** Comparison of ^13^C NMR chemical shifts in thymine incubated in concentrated sulfuric acid for two different time periods. All NMR data were obtained at room temperature.

Thymine										
^13^C										
Solvent	C2 (ppm)		C4 (ppm)		C5 (ppm)		C6 (ppm)		CH_3_ (ppm)	
	*13 October 2022*	*7 November 2023*	*13 October 2022*	*7 November 2023*	*13 October 2022*	*7 November 2023*	*13 October 2022*	*7 November 2023*	*13 October 2022*	*7 November 2023*
81% D_2_SO_4_/19% D_2_O	146.93	146.73	167.79	167.77	107.89	107.95	149.61	149.68	10.41	10.42
88% D_2_SO_4_/12% D_2_O	148.27	148.17	167.88	167.87	107.68	107.68	149.18	149.21	10.26	10.26
94% D_2_SO_4_/6% D_2_O	148.97	148.98	167.92	167.92	107.83	107.81	149.08	149.02	10.12	10.12
98% D_2_SO_4_/2% D_2_O	148.95	148.95	167.98	167.97	108.09	108.07	149.59	149.51	9.99	10.00

**Table 5:** Comparison of ^13^C NMR chemical shifts in adenine incubated in concentrated sulfuric acid for two different time periods. All NMR data were obtained at room temperature.

Adenine										
^13^C										
Solvent	C2 (ppm)		C4 (ppm)		C5 (ppm)		C6 (ppm)		C8 (ppm)	
	*13 October 2022*	*6 November 2023*	*13 October 2022*	*6 November 2023*	*13 October 2022*	*6 November 2023*	*13 October 2022*	*6 November 2023*	*13 October 2022*	*6 November 2023*
81% D_2_SO_4_/19% D_2_O	147.92	147.90	141.78	141.76	108.61	108.60	146.67	146.67	146.11	146.15
88% D_2_SO_4_/12% D_2_O	148.18	148.13	142.01	141.96	108.74	108.71	146.59	146.61	145.00	145.22
94% D_2_SO_4_/6% D_2_O	148.08	148.03	141.97	141.94	108.71	108.68	146.65	146.66	145.50	145.70
98% D_2_SO_4_/2% D_2_O	149.55	149.47	137.37	137.78	110.22	110.14	146.43	146.45	143.42	143.35

**Table 6:** Comparison of ^13^C NMR chemical shifts in uracil incubated in concentrated sulfuric acid for two different time periods. All NMR data were obtained at room temperature.

Uracil								
^13^C								
Solvent	C2 (ppm)		C4 (ppm)		C5 (ppm)		C6 (ppm)	
	*13 October 2022*	*7 November 2023*	*13 October 2022*	*7 November 2023*	*13 October 2022*	*7 November 2023*	*13 October 2022*	*7 November 2023*
81% D_2_SO_4_/19% D_2_O	141.52	141.62	162.69	162.72	88.50	88.59	145.19	145.11
88% D_2_SO_4_/12% D_2_O	148.40	148.43	169.68	169.63	95.51	95.50	152.56	152.43
94% D_2_SO_4_/6% D_2_O	148.42	148.42	169.60	169.57	95.74	95.71	152.82	152.70
98% D_2_SO_4_/2% D_2_O	148.41	148.43	169.48	169.49	95.93	95.93	153.07	152.96

**Table 7:** Comparison of ^13^C NMR chemical shifts in purine incubated in concentrated sulfuric acid for two different time periods. All NMR data were obtained at room temperature.

Purine										
^13^C										
Solvent	C2 (ppm)		C4 (ppm)		C5 (ppm)		C6 (ppm)		C8 (ppm)	
	*11 October 2022*	*7 November 2023*	*11 October 2022*	*7 November 2023*	*11 October 2022*	*7 November 2023*	*11 October 2022*	*7 November 2023*	*11 October 2022*	*7 November 2023*
81% D_2_SO_4_/19% D_2_O ^1^	149.28	149.27	151.44	151.44	121.09	121.09	140.49	140.48	150.14	150.14
88% D_2_SO_4_/12% D_2_O	149.36	149.34	151.30	151.30	120.92	120.93	140.57	140.55	150.12	150.11
94% D_2_SO_4_/6% D_2_O	149.39	149.39	151.16	151.18	120.78	120.79	140.61	140.61	150.06	150.07
98% D_2_SO_4_, 2% D_2_O	149.39	149.39	151.04	151.04	120.66	120.68	140.66	140.65	150.00	150.01

^1^ After the one-year-long incubation of purine in 81% *w*/*w* sulfuric acid, one new peak emerges at 167.66 ppm. The new peak likely corresponds to an unknown reactive contaminant in the reaction mixture and does not indicate the instability of a purine ring, as all chemical shifts of the purine ring remain unchanged. See also https://zenodo.org/records/10793625 (accessed on 7 March 2024).

**Table 8:** Comparison of ^13^C NMR chemical shifts in pyrimidine incubated in concentrated sulfuric acid for two different time periods. All NMR data were obtained at room temperature.

Pyrimidine						
^13^C						
Solvent	C2 (ppm)		C4,6 (ppm)		C5 (ppm)	
	*13 October 2022*	*7 November 2023*	*13 October 2022*	*7 November 2023*	*13 October 2022*	*7 November 2023*
81% D_2_SO_4_/19% D_2_O	143.79	143.89	150.80	150.81	117.91	117.91
88% D_2_SO_4_/12% D_2_O	150.55	150.57	157.64	157.55	124.92	124.82
94% D_2_SO_4_/6% D_2_O	150.04	150.05	158.01	157.99	127.06	126.97
98% D_2_SO_4_, 2% D_2_O	149.85	149.86	158.18	158.17	127.77	127.73

## Data Availability

Original data are deposited in the Zenodo data repository at https://zenodo.org/records/10793625 (accessed on 7 March 2024). The authors are also willing to provide the original datasets upon request. To request the raw data, please contact Janusz J. Petkowski (jjpetkow@mit.edu).

## References

[1] Morowitz H., Sagan C. (1967). Life in the clouds of venus?. Nature.

[2] Patel M.R., Mason J.P., Nordheim T.A., Dartnell L.R. (2021). Constraints on a potential aerial biosphere on Venus: II. Ultraviolet radiation. Icarus.

[3] Mogul R., Limaye S.S., Lee Y.J., Pasillas M. (2021). Potential for Phototrophy in Venus’ Clouds. Astrobiology.

[4] Kotsyurbenko O.R., Cordova J.A., Belov A.A., Cheptsov V.S., Kölbl D., Khrunyk Y.Y., Kryuchkova M.O., Milojevic T., Mogul R., Sasaki S. (2021). Exobiology of the Venusian Clouds: New Insights into Habitability through Terrestrial Models and Methods of Detection. Astrobiology.

[5] Limaye S.S., Mogul R., Smith D.J., Ansari A.H., Słowik G.P., Vaishampayan P. (2018). Venus’ Spectral Signatures and the Potential for Life in the Clouds. Astrobiology.

[6] Schulze-Makuch D., Irwin L.N. (2006). The prospect of alien life in exotic forms on other worlds. Naturwissenschaften.

[7] Grinspoon D.H., Bullock M.A. (2007). Astrobiology and Venus exploration. Geophys. Monogr. Geophys. Union.

[8] Seager S., Petkowski J.J., Gao P., Bains W., Bryan N.C., Ranjan S., Greaves J. (2021). The Venusian Lower Atmosphere Haze as a Depot for Desiccated Microbial Life: A Proposed Life Cycle for Persistence of the Venusian Aerial Biosphere. Astrobiology.

[9] Bains W., Petkowski J.J., Seager S. (2024). Venus’ atmospheric chemistry and cloud characteristics are compatible with Venusian life. Astrobiology.

[10] Wagner R., von Philipsborn W. (1970). Protonierung von Amino-und Hydroxypyrimidinen NMR-Spektren und Strukturen der Mono-und Dikationen. Helv. Chim. Acta.

[11] Schumacher M., Günther H. (1983). Beiträge zur 15N-NMR-Spektroskopie Protonierung und Tautomerie in Purinen: Purin und 7-und 9-Methylpurin. Chem. Ber..

[12] Wagner R., von Philipsborn W. (1971). Protonierung von Purin, Adenin und Guanin NMR.-Spektren und Strukturen der Mono-, Di-und Tri-Kationen. Helv. Chim. Acta.

[13] Albert A., Brown D.J. (1954). Purine studies. Part I. Stability to acid and alkali. Solubility. Ionization. Comparison with pteridines. J. Chem. Soc..

[14] Seager S., Petkowski J.J., Seager M.D., Grimes J.H., Zinsli Z., Vollmer-Snarr H.R., Abd El-Rahman M.K., Wishart D.S., Lee B.L., Gautam V. (2023). Stability of nucleic acid bases in concentrated sulfuric acid: Implications for the habitability of Venus’ clouds. Proc. Natl. Acad. Sci. USA.

[15] Willcott M.R. (2009). MestRe Nova. J. Am. Chem. Soc..

[16] Dračínský M., Holý A., Jansa P., Kovačková S., Buděšínský M. (2009). Isotopic Exchange of Hydrogen at C-5 in Pyrimidine Derivatives: Tautomers with an sp3-Hybridised C-5 Carbon Atom. Eur. J. Org. Chem..

[17] Bains W., Petkowski J.J., Zhan Z., Seager S. (2021). Evaluating Alternatives to Water as Solvents for Life: The Example of Sulfuric Acid. Life.

[18] Bains W., Petkowski J.J., Seager S. (2021). A Data Resource for Sulfuric Acid Reactivity of Organic Chemicals. Data.

[19] Oba Y., Takano Y., Furukawa Y., Koga T., Glavin D.P., Dworkin J.P., Naraoka H. (2022). Identifying the wide diversity of extraterrestrial purine and pyrimidine nucleobases in carbonaceous meteorites. Nat. Commun..

[20] Rotelli L., Trigo-Rodríguez J.M., Moyano-Cambero C.E., Carota E., Botta L., Di Mauro E., Saladino R. (2016). The key role of meteorites in the formation of relevant prebiotic molecules in a formamide/water environment. Sci. Rep..

[21] Ge P., Luo G., Luo Y., Huang W., Xie H., Chen J. (2018). A molecular-scale study on the hydration of sulfuric acid-amide complexes and the atmospheric implication. Chemosphere.

